# Growth and Maximum Size of Tiger Sharks (*Galeocerdo cuvier*) in Hawaii

**DOI:** 10.1371/journal.pone.0084799

**Published:** 2014-01-08

**Authors:** Carl G. Meyer, Joseph M. O'Malley, Yannis P. Papastamatiou, Jonathan J. Dale, Melanie R. Hutchinson, James M. Anderson, Mark A. Royer, Kim N. Holland

**Affiliations:** 1 Hawaii Institute of Marine Biology, University of Hawaii at Manoa, Honolulu, Hawai'i, United States of America; 2 National Marine Fisheries Service, Pacific Islands Fisheries Science Center, Honolulu, Hawai'i, United States of America; 3 Florida Museum of Natural History, University of Florida, Gainesville, Florida, United States of America; 4 School of Biology, Scottish Oceans Institute, University of St Andrews, St Andrews, Scotland, United Kingdom; 5 Hopkins Marine Station, Stanford University, Pacific Grove, California, United States of America; University of Sussex, United Kingdom

## Abstract

Tiger sharks (*Galecerdo cuvier*) are apex predators characterized by their broad diet, large size and rapid growth. Tiger shark maximum size is typically between 380 & 450 cm Total Length (TL), with a few individuals reaching 550 cm TL, but the maximum size of tiger sharks in Hawaii waters remains uncertain. A previous study suggested tiger sharks grow rather slowly in Hawaii compared to other regions, but this may have been an artifact of the method used to estimate growth (unvalidated vertebral ring counts) compounded by small sample size and narrow size range. Since 1993, the University of Hawaii has conducted a research program aimed at elucidating tiger shark biology, and to date 420 tiger sharks have been tagged and 50 recaptured. All recaptures were from Hawaii except a single shark recaptured off Isla Jacques Cousteau (24°13′17″N 109°52′14″W), in the southern Gulf of California (minimum distance between tag and recapture sites  =  approximately 5,000 km), after 366 days at liberty (DAL). We used these empirical mark-recapture data to estimate growth rates and maximum size for tiger sharks in Hawaii. We found that tiger sharks in Hawaii grow twice as fast as previously thought, on average reaching 340 cm TL by age 5, and attaining a maximum size of 403 cm TL. Our model indicates the fastest growing individuals attain 400 cm TL by age 5, and the largest reach a maximum size of 444 cm TL. The largest shark captured during our study was 464 cm TL but individuals >450 cm TL were extremely rare (0.005% of sharks captured). We conclude that tiger shark growth rates and maximum sizes in Hawaii are generally consistent with those in other regions, and hypothesize that a broad diet may help them to achieve this rapid growth by maximizing prey consumption rates.

## Introduction

Tiger sharks (*Galeocerdo cuvier*) are wide-ranging [1]–[4] apex predators that consume a diverse array of vertebrate and invertebrate prey [5],[6], while utilizing a broad variety of coastal and oceanic habitats [1]–[4]. They reproduce on a triennial cycle with average litter sizes of 30 to 50 pups ranging in size from 80 to 90 cm Total Length (TL) at birth [7]. Tiger sharks grow to a large size, with 13 studies published between 1975 and 2012 reporting maximum TL ranging from 381 to 550 cm (Table 1), however, an earlier, unconfirmed report suggested that female tiger sharks can reach 740 cm TL [8]. Previous studies indicate they grow relatively fast (*K* = 0.11– 0.46 year^−1^; [9]–[12]) compared to some other carcharhinid sharks (e.g. *K* = 0.10 – 0.12 year^−1^ for Hawaii sandbar sharks, *Carcharhinus plumbeus*; [14]). There is, however, considerable regional variation in reported tiger shark growth rates. For example, juvenile tiger sharks grow almost twice as fast in the Gulf of Mexico as in the Northwest Atlantic [9]. Some of this variation may stem from the different methods used to estimate growth (e.g. vertebral ring counts versus mark-recapture experiments) [15], compounded by small sample sizes and lack of validation in some studies [16]. The only study specifically examining tiger shark growth in Hawaii, suggests that growth is relatively slow (*K* = 0.16 year^−1^, [16]) compared to several other regions (e.g. *K* = 0.2 year^−1^ in South Africa, [11]; *K* = 0.27–0.46 in NW Atlantic, [13]). However, the veracity of the existing Hawaii growth estimates are questionable given the lack of validation of vertebral annual ring formation [17], overall small sample size (N = 28), and limited data for small and large size classes.

**Table 1:** Empirically measured tiger shark maximum sizes from peer-reviewed literature.

Region	Maximum size (cm TL) [sex]	N	Source of sample	Reference [citation]
Australia (Queensland)	550 [Female]	4757	Shark control program, animals measured by commercial contractors	Holmes et al. 2012 [41]
Australia (Queensland)	428 [Female]	835	Shark control program, animals measured by commercial contractors	Simpfendorfer 1992 [40]
Australia (Northern Australia)	418 [Female]	299	Commercial gill-net fisheries and scientific research cruises (long line, trawl), animals measured by fisheries observers and scientific personnel	Stevens and McLoughlin 1991 [39]
Australia (New South Wales)	382 [Male]	89	Sportfishing catches, some lengthsmeasured by scientific personnel others derived from weight using Length-Weight relationships established by Stevens 1984	Stevens 1984 [48]
Australia (Western Australia)	445 [Not given]	449	Scientific study using single-hook drumlines	Wirsing et al. 2006 [12]
Australia (Western Australia)	407 [Not given]	252	Scientific study using single-hook drumlines	Heithaus 2001 [46]
Australia (Western Australia)	430 [Female]	225	Scientific study using single, or double-hook setlines	Simpfendorfer et al. 2001 [6]
South Africa (KwaZulu-Natal)	410 [Female]	54	Shark control program and commercial fisheries, measured by scientific personnel	Bass et al. 1975 [47]
South Africa (KwaZulu-Natal)	392 [Male]	101	Shark control program measured by scientific personnel	Wintner and Dudley 2000 [11]
USA (Western North Atlantic)	417 [Female]	238	Research cruises, commercial and recreational fishing vessels, sportfishing tournaments, measured by scientific personnel	Kneebone et al. 2008 [13]
USA (Atlantic/Gulf of Mexico)	381 [Male and Female]	163 (1)	Commercial and research longline catches, recreational tournament catches, measured by scientific personnel	Branstetter et al. 1987 [48]
USA (Gulf of Mexico)	410 [Female]	45	Longline and sportfishing catches measured by scientific personnel	Branstetter 1981 [49]
USA (Hawaii)	447 [Female]	318	Shark control program, incidental and research catches measured by scientific personnel	Whitney and Crow 2007 [7]

For ease of comparison, all measurements are Total Length in cm. Where necessary, original PCL of FL values have been converted to TL using length-length conversion relationships given in Table 3.

Unlike vertebral ring counts where growth is inferred rather than measured, mark-recapture methods empirically measure growth of animals between two points in time [12],[18],[19]. However, obtaining sufficient mark-recapture data to generate accurate growth curves for large marine predators is challenging because a sample size of hundreds of tagged animals representive of the species entire size range is generally required to yield a sufficient number of recaptures (recapture rates for tiger sharks are typically <10%; [10]). Furthermore, long periods of time (months or years) are needed for growth to outpace the potential influence of measurement errors. Thus, a sustained effort over many years is generally necessary to generate robust estimates of growth for species such as tiger sharks. For twenty years, the University of Hawaii has conducted a research program aimed at elucidating tiger shark biology, and throughout this period tiger sharks have been tagged and periodically recaptured. Here we use these empirical mark-recapture data to estimate growth rates and maximum size for tiger sharks in Hawaii.

## Materials and Methods

### Ethics Statement

Vertebrate work carried out during this study was approved by the University of Hawaii Institutional Animal Care and Use Committee (protocol #05-053 et seq.). Field studies did not involve endangered or protected species. Sharks were captured in both Hawaii state waters, where no specific permissions were required, and under permit in the Papahanaumokuakea Marine National Monument (U.S. Fish and Wildlife Special Use Permit #12521-06048, State of Hawaii Department of Land and Natural Resources permits # DLNR.NWHI06R019, NOAA- NWHIMNM- permit #2006-012, and Papahanaumokuakea Marine National Monument permits # PMNM-2007-031, #PMNM-2008-027 and # PMNM-2009-037).

### Capture and tagging

From September 1993 to January 2013, we captured tiger sharks at various locations throughout the Hawaiian Archipelago. Sharks were captured using demersal long-lines baited with large tuna heads and fish scraps, and soaked for 2–12 h in depths of 10 to 100 m [21]. Captured sharks were brought alongside a 6 m skiff, where they were tail-roped and inverted to initiate tonic immobility. Three length measurements were recorded from each shark; Pre-caudal Length (PCL- tip of the snout to the pre-caudal pit), Fork Length (FL- tip of the snout to the caudal fork), and Total Length (TL- tip of the snout to a point on the horizontal axis intersecting a perpendicular line extending down from the tip of the upper caudal lobe) (for illustrated details of shark measurement protocols see [22]). Sex was determined by the presence, size and degree of calcification of claspers. Sharks were tagged using either NMFS ‘M’ capsule or Hallprint™ ‘spaghetti’ shark tags inserted into the dorsal musculature at the base of the first dorsal fin. The NMFS ‘M’ tags consist of a stainless steel dart head, connected via monofilament line to a Plexiglas capsule containing waterproof paper printed with the tag number and return instructions (see also [20]). The Hallprint™ ‘spaghetti’ shark tags (Hallprint Pty. Ltd., Victor Harbor, South Australia) were of the 'wire through' variety (plastic sheath surrounding a stainless steel wire core connected to the stainless steel dart head) with contact details and a unique ID number printed along the plastic sheath. We switched from NMFS capsule tags to Hallprint tags because several capsule-tagged sharks were recaptured with the plastic data capsules missing.

### Analyses of Size and Growth

We used a one-way ANOVA with least squares testing of means to compare the average sizes of all captured male and female tiger sharks. Linear regression analyses were used to generate length-to-length conversion formulae (i.e. PCL to FL etc.) from our data set (both sexes combined). A Chi-squared test was used to determine whether tiger shark sex ratio was significantly different from 1∶1.

Sex-specific and combined-sex mark-recapture data were fitted to the von Bertalanffy growth equation using two different methods. The first utilized the Gulland and Holt [23] method which uses linear regression to fit a line through a plot of average size versus annual growth rates with *-K* equaling the slope of the line and *L_∞_* equaling the *x*-intercept. The second method was an adaptation of the Francis [18] maximum likelihood model (GROTAG) for the Microsoft Excel solver function [19]. This method is a re-parameterization of the Fabens' (1965) growth model, where the usual von Bertalanffy parameters, *K* and *L_∞_*, are replaced by two alternative parameters, *g*_α_ and *g*_β_, which represent mean annual growth increments (mm/yr) of chosen reference lengths *α* and *β* [18]. These parameters have better statistical properties than *K* and *L_∞_*, particularly when the entire size range of the species is not represented in the data [15], [18]. Additional parameters, such as growth variability (*v*), the mean (*m*) and standard deviation (*s*) of measurement error, seasonality (*u*, *w*), and outlier contamination (*p*) were added to the model in a step-wise fitting procedure. Likelihood ratio tests (LRT) were used to determine the final model, where for a significant (*P*<0.05) improvement in fit, the likelihood value must increase by at least 1.92 with the introduction of one parameter and 3.0 with the introduction of two parameters [18]. Ninety-five percent confidence intervals (CIs) were estimated using a bootstrapping method as implemented in GROTAG [19]. Significant differences between data sets were identified by comparing CIs and the extent of bootstrap overlap [24].

Only data from sharks at liberty for more than 25 days and with positive growth rates were used to generate growth estimates. Tiger shark reference lengths, *α* (104 cm) and *β* (240 cm) were chosen so that the majority of individuals in each data set were between these two values [18]. To facilitate comparisons of Hawaiian tiger shark growth parameters with those from previous studies [9], [10], [12] we used PCL measurements as the basis for our growth rate estimates and the GROTAG model outputs were converted to the von Bertalanffy growth parameters *K* and *L_∞_* following Francis [18]. The best fit von Bertalanffy growth parameters *K* and *L_∞_* derived from the GROTAG model were used to generate a growth curve for Hawaii tiger sharks (both sexes combined) assuming a birth size of 85 cm TL [7].

## Results

**Figure 1:**
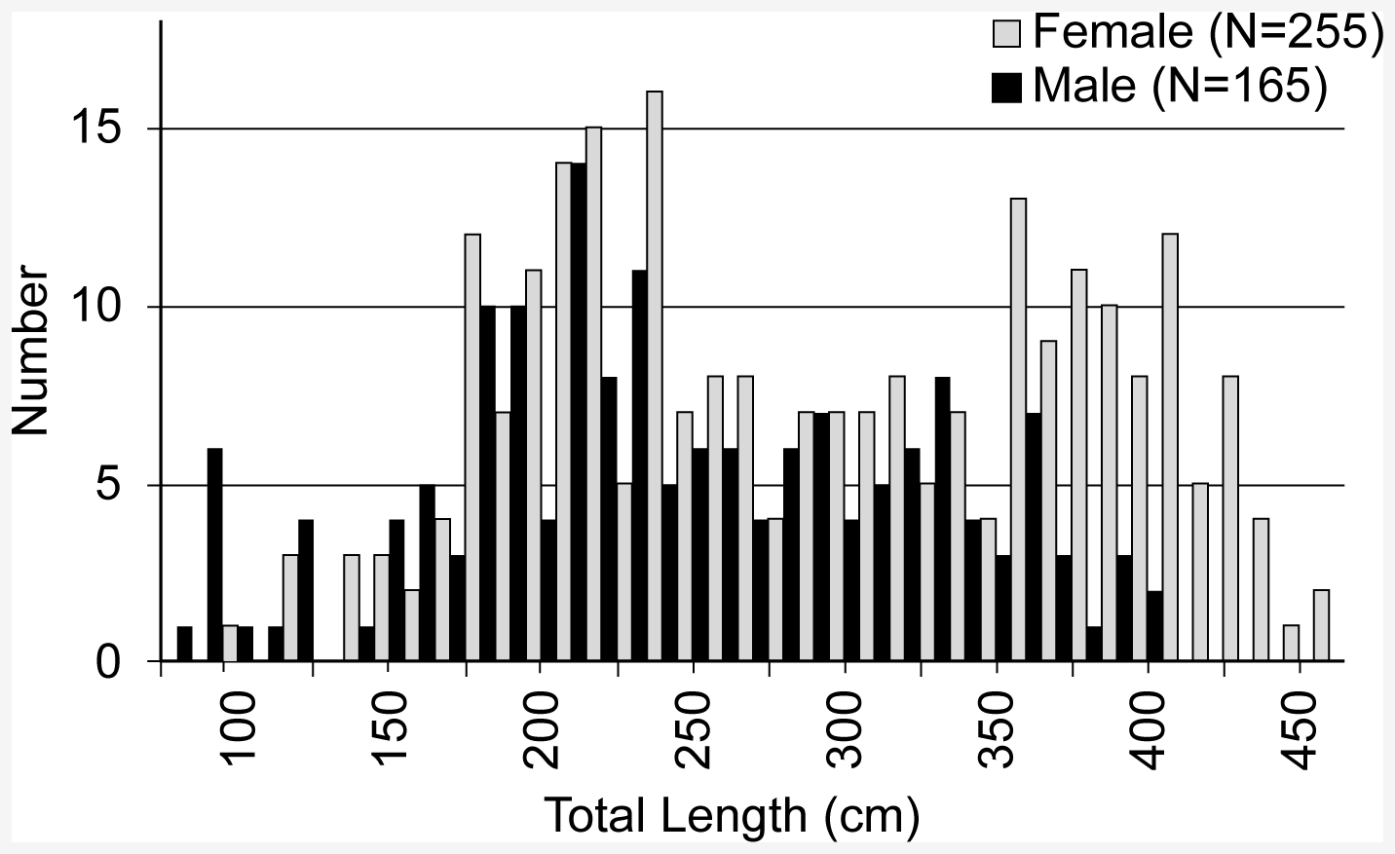
Size distribution of male and female tiger sharks captured in Hawaii 1993–2013.

Four hundred and twenty tiger sharks ranging in size from 88 to 464 cm TL (57 to 365 cm PCL) were captured from 1993 to 2013 (Fig. 1). The sex ratio of our sample was significantly skewed toward females (male:female ratio  = 0.65, χ^2^ = 21.7, df = 1, p<0.001), and females were significantly larger on average than males (Table 2). The largest male tiger shark in our sample was 406 cm TL (327 cm PCL), whereas the largest female was 464 cm TL (365 cm PCL), and 14% of females captured were larger than the biggest male captured (Fig. 1). Overall, tiger sharks of at least 400 cm TL (∼306 cm PCL) were relatively common, accounting for 10% of our sample, but individuals larger than 450 cm TL (∼347 cm PCL) were very rare, accounting for only 2 of 420 (0.005%) sharks captured (Fig. 1). Length-length regression analyses yielded conversion relationships with R^2^ values >0.98 (Table 3).

**Table 2:** Average size of male and female tiger sharks captured by research fishing in Hawaii 1993–2013.

	Male	Female	F	df	p
Total length	248.5 (84)[163]	296.8 (84)[251]	33.0	1, 412	p<0.001
Fork length	201.5 (73)[164]	243.1 (73)[249]	32.3	1, 411	p<0.001
Precaudal length	183.2 (68)[164]	220.9 (68)[253]	30.7	1, 415	p<0.001

Numbers in parentheses are standard deviations, numbers in square brackets are sample sizes. Anova test statistics (F) for the comparison of mean male and female sizes are given along with degrees of freedom (df) and probability values (p).

**Table 3:** Length to length relationships for tiger sharks (male and female combined) captured in Hawaii 1993–2013.

x	y	b_0_	b_1_	r^2^
PCL	TL	26·15 (1·78)	1·22 (0·008)	0·98
TL	PCL	−17·38 (1·57)	0·81 (0·005)	0·98
FL	TL	19·62 (1·68)	1·14 (0·007)	0·99
TL	FL	−13·57 (1.56)	0·87 (0·005)	0·99
PCL	FL	6·07 (0·68)	1·07 (0·003)	0·99
FL	PCL	−4·95 (0·64)	0·93 (0·003)	0·99

Linear regression coefficients are for the model *y*_i_ = *b*_0_+*b*_1_ *x*_i_. s.e. of the means are in parenthesis.

PCL, Pre-caudal Length (cm); FL, Fork Length (cm); TL, Total Length (cm).

Of the 420 tiger sharks tagged and released, 50 (11.9%) were recaptured, each on 1–3 occasions. For recaptured sharks, size at release ranged from 96 to 373 cm TL (63 to 282 cm PCL), size at recapture ranged from 174 to 384 cm TL (127 to 290 cm PCL) and time at liberty ranged from 15 to 2,421 days (median  = 197 days). All recaptures were from within the Hawaiian Archipelago except a single shark recaptured by Mexican fishermen off Isla Jacques Cousteau (formerly Isla Cerralvo, 24°13′17″N 109°52′14″W), in the southern Gulf of California (minimum distance between tag and recapture sites  =  approximately 5,000 km), after 366 days at liberty (DAL). Recaptured sharks with damaged or unreadable tags (N = 7), those not measured by scientific personnel (N = 2) or with negative growth (N = 4), were removed from the data set. The remaining 26 females, 10 males, and an additional unsexed individual were used in the sex-specific and sexes combined growth analyses (Table 4).

**Table 4:** Details of recaptured sharks used to estimate growth models.

Sex	Tag Date	Recapture Date	Days at Liberty	PCL-1 (cm)	PCL-2 (cm)	Growth overall PCL (cm)	Annual growth rate PCL (cm/y)
F	10/26/11	11/21/12	392	108	131	23	21.4
F	1/30/09	3/16/09	45	117	127	10	81.1
F	3/9/09	6/3/09	86	128	150	22	93.4
F	6/29/01	11/18/02	507	131	182	51	36.7
F	1/12/05	3/3/09	1511	136	256	120	29.0
F	10/12/95	2/27/96	138	138	161	23	60.8
F	6/11/11	12/6/11	178	140	170	30	61.5
F	6/8/08	6/15/09	372	150	188	38	37.3
F	2/23/01	8/24/01	182	151	176	25	50.1
F	6/20/08	11/17/09	515	159	222	63	44.7
F	11/16/95	10/16/00	1796	160	277	117	23.8
F	10/24/95	1/30/96	98	164	179	15	55.9
F	3/7/02	11/18/02	256	164	193	29	41.3
F	4/16/09	4/10/12	1090	170	245	75	25.1
F	8/24/01	3/7/02	195	178	193	15	28.1
F	3/7/02	5/22/02	76	179	185	6	28.8
F	3/7/02	9/4/02	181	181	204	23	46.4
F	8/24/01	11/18/02	451	202	255	53	42.9
F	9/10/96	8/12/97	336	208	219	11	11.9
F	8/3/08	5/26/09	296	210	221	11	13.6
F	3/16/09	4/16/09	31	212	215	3	35.3
F	2/19/08	7/21/11	1248	214	249	35	10.2
F	1/10/95	5/19/95	129	224	235	11	31.1
F	8/9/96	11/6/96	89	233	235	2	8.2
F	12/7/94	5/10/95	154	249	251	2	4.7
F	8/24/08	7/21/09	331	280	290	10	11.0
M	12/11/10	8/25/11	257	63	138	75	106.5
M	6/29/01	2/14/08	2421	136	257	121	18.2
M	5/10/94	11/16/95	555	162	218	56	36.8
M	10/1/96	10/29/96	28	164	168	4	52.1
M	8/24/01	11/18/02	451	177	191	14	11.3
M	10/26/08	12/2/09	402	221	241	20	18.2
M	10/12/94	10/24/95	377	224	245	21	20.3
M	8/10/95	2/2/96	176	229	237	8	16.6
M	1/20/95	5/10/95	110	240	248	8	26.5
M	5/19/95	12/5/95	200	269	278	9	16.4
U	10/8/10	9/26/11	353	263	280	17	17.6

PCL  =  Precaudal Length.

### Gulland and Holt (1959) method

Linear regression analyses of average PCL versus annual growth rates yielded negative slopes (i.e. declining growth with increasing length) for males, females and both sexes combined. Female *K* was 0.40 and *PCL_∞_* was 283.2 cm (*TL_∞_*  =  ∼372 cm), male *K* was 0.48 and *PCL_∞_* was 272.1 cm (*TL_∞_*  =  ∼358 cm), and overall *K* was 0.41 and *PCL_∞_* was 282.5 cm (*TL_∞_*  =  ∼371 cm).

### Francis (1988a) method

The models containing *ga*, *gb*, growth variability, and standard deviation of measurement error resulted in the best fit to the von Bertalanffy growth model for all data sets (Table 5). Model fit was not significantly improved by the inclusion of additional parameters, as evident in the likelihood ratio tests. However, with the exception of model 1 for male and model 4 for female tiger sharks, estimates of all parameters were very similar among all models and all data sets. Similarity between parameter estimates and the low value of the standard deviation of measurement error suggests the model was unable to accurately estimate measurement error, probably due to low sample size and lack of individuals at liberty for short amounts of time (<30 days) in the final data set. No individuals had absolute standardized residuals greater than 3.0; therefore, exclusion of the outlier contamination parameter from the final model was warranted as also evident by the lack of improvement of fit with its inclusion.

**Table 5:** Growth models and parameter values.

			Parameter estimates										
Sex	Model	Likelihood	*g_108_*	*g_240_*	*v*	*s*	*m*	*p*	*u*	*w*	*k*	*PCL_∞_*	*TLL_∞_*
			(cm/yr)	(cm/yr)		(cm)	(cm)			(yr)		(cm)	*(cm)*
male	1	−40.3	61.48	13.74	-	5.84	-	-	-	-	0.45	278.00	365.31
	**2**	−**34.01**	**54.61 (35.44–73.86)**	**19.11 (13.23–24.63)**	**0.34 (0.12–0.47)**	**0.002 (0–2.98)**	**-**	**-**	**-**	**-**	**0.31 (0.12–0.55)**	**311.04 (275.52–404.96)**	**405.62 (362.28–520.20)**
	3	−33.35	47.80	16.08	0.37	0.0008	1.61	-	-	-	0.28	306.91	400.58
	4	−33.35	47.80	16.08	0.37	0.0008	1.61	0	-	-	0.28	306.91	400.58
	5	−33.18	58.76	19.41	0.31	0.23	-	-	0.56	0.47	0.35	305.13	398.41
female	1	−96.83	49.52	15.10	-	8.98	-	-	-	-	0.30	297.93	389.63
	**2**	**−92.13**	**53.37 (44.78–62.84)**	**18.30 (14.32–22.26)**	**0.38 (0.23–0.48)**	**0.66 (0–2.73)**	**-**	**-**	**-**	**-**	**0.31 (0.22–0.42)**	**308.90 (285.57–342.80)**	**403.01 (374.55–444.37)**
	3	−92.11	52.66	17.99	0.38	0.75	0.20	-	-	-	0.31	308.55	402.58
	4	−99.04	49.46	11.50	0.52	0.51	0.53	0.26	-	-	0.34	280.00	367.75
	5	−91.39	51.83	17.96	0.38	0.000005	-	-	0.19	0.23	0.30	309.99	404.34
both	1	−141.06	53.41	14.67	-	7.99	-	-	-	-	0.35	290.00	379.95
	**2**	**−129.18**	**53.90 (45.90–62.38)**	**18.93 (15.91–21.77)**	**0.37 (0.24–0.44)**	**0 (0–2.21)**	**-**	**-**	**-**	**-**	**0.31 (0.23–0.40)**	**311.47 (292.69–336.14)**	**406.14 (383.23–436.24)**
	3	−128.90	51.43	17.93	0.38	0	0.61	-	-	-	0.29	310.60	405.08
	4	−128.90	51.43	17.93	0.38	0	0.61	0	-	-	0.29	310.60	405.08
	5	−128.33	53.24	18.88	0.36	0	-	-	0.14	0.29	0.30	312.55	407.46

Parameter estimates and log-likelihood values estimated using the Francis (1988a) method for tiger shark sex-specific and both sexes combined data sets. Bold text indicates optimally parameterized model for each data set. Numbers in parentheses are bootstrap 95% confidence intervals for the best fit models. PCL ∞ was converted to TL∞ using coefficients in Table 3. Different models represent step-wise inclusions of further parameters. Abbreviations as follows: g108  =  mean annual growth increment at 108 cm, g240  =  mean annual growth increment at 240 cm, v  =  coefficient of variation of growth variability, s  =  standard deviation of measurement error, m  =  mean measurement error, p  =  outlier contamination, u  =  seasonality amplitude, w  =  time of year when growth is at maximum, k  =  von Bertalanffy growth constant, PCL∞  =  asymptotic maximum precaudal length, TLL∞  =  asymptotic maximum total length. The different models represent step-wise inclusions of parameters.

**Figure 2:**
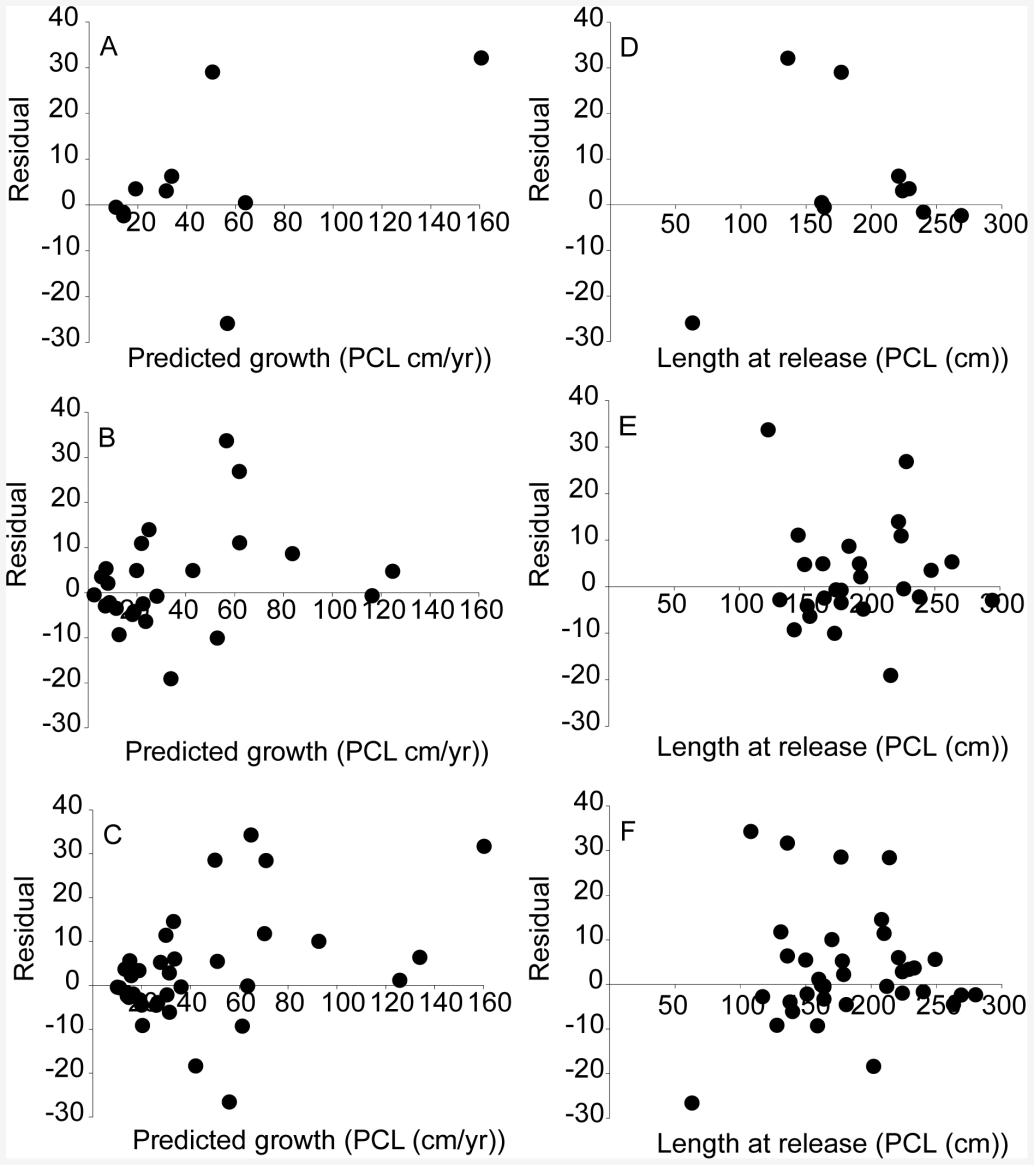
Francis growth model residual plots. Plots of Francis growth model residuals (observed minus predicted) versus predicted growth (PCL (cm/yr)) for tiger shark a) male, b) female, and c) sexes combined and length-at-release (PCL (cm)) for d) male, e) female, and f) sexes combined.

To assess final model fits, residuals were plotted against length-at-release and predicted growth (Figure 2). Residuals declined with increasing length-at-release because mean growth declined with length [25], whereas residuals versus predicted growth showed the opposite trend, as would be expected. Standardized residuals (residuals divided by σ*_i_*, which, in the selected models, equals *s*) showed no pattern, indicating that the model assumption that growth variability is dependent on mean growth was not violated [15]. Overall, suitability of final models from tiger shark mark-recapture data was indicated by residual plots.

The best fit models for the sex-specific and both sexes combined data sets resulted in very similar growth-at-size estimates (*ga*, *gb*) and the converted *K* and *L_∞_* (Table 5). The estimated *K* was 0.31 for all data sets and *PCL_∞_* ranged from 308.9 cm (*TL_∞_*  =  ∼403.0 cm) to 311.5 cm (*TL_∞_*  =  ∼405.8 cm). The estimated growth variability ranged from 0.34 to 0.38 between data sets. Ninety-five percent confidence intervals were greater for males relative to females and both sexes combined, likely due to the lower sample size of males.

**Figure 3:**
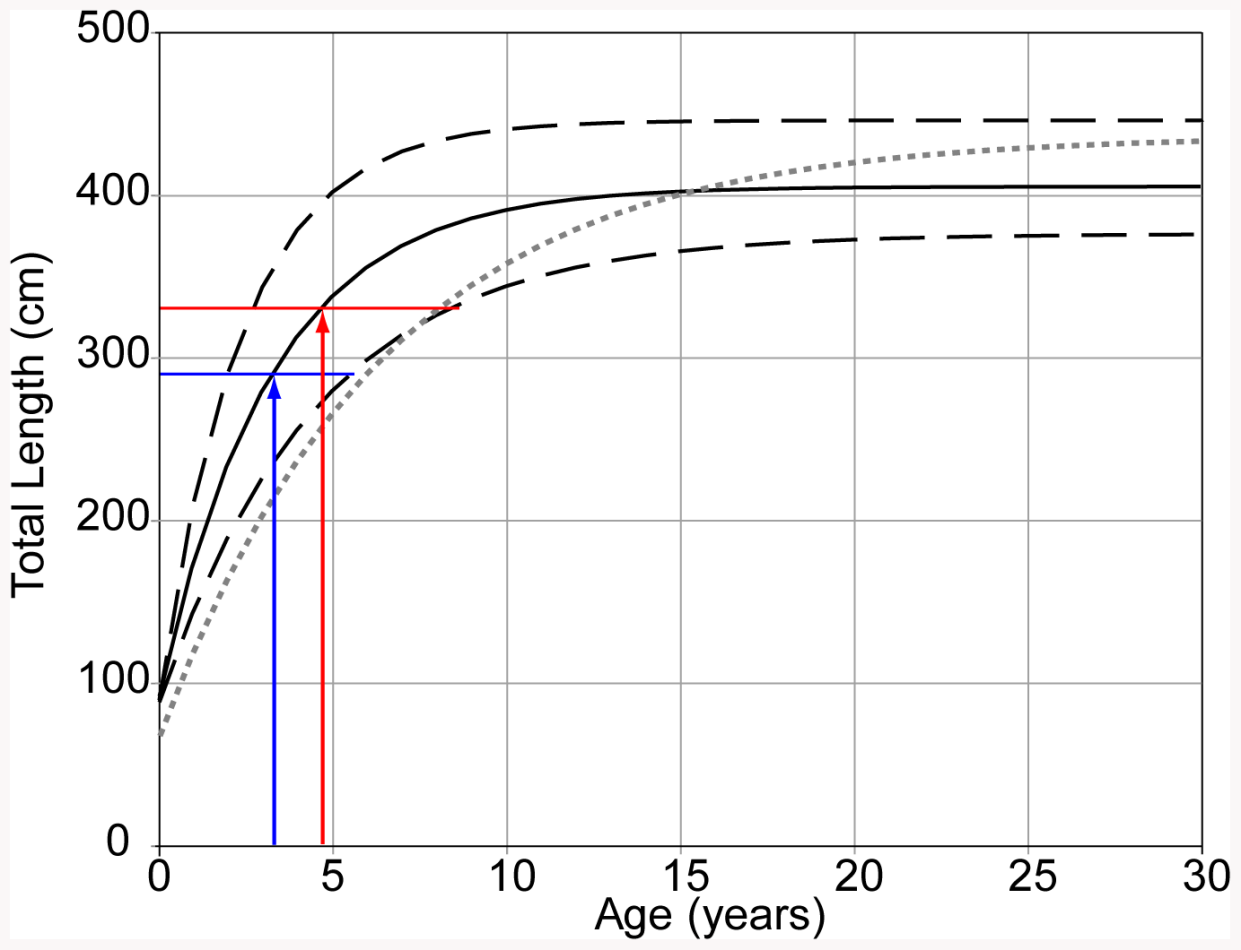
Von Bertalanffy growth curves for Hawaii tiger sharks (both sexes combined). Growth curves are derived from parameters generated by the best fit GROTAG model (Solid line  =  average growth. Dashed line  =  upper and lower 95% growth estimates) and analysis of vertebral rings (De Crosta et al. [16], dotted grey line) Arrows indicate estimates of minimum age at maturity for male (blue) and female (red) tiger sharks based on size at maturity estimates (horizontal lines) provided by Whitney and Crow (2007) [7].

The growth curve generated from Von Bertalanffy parameters produced by the best fit GROTAG model (Figure 3) illustrates the early rapid growth of tiger sharks in Hawaii. On average, tiger sharks in Hawaii reach 340 cm TL by age 5, and attain a maximum size of 403 cm TL. Our model further indicates that the fastest growing individuals attain 400 cm TL by age 5, and the largest reach a maximum size of 444 cm TL.

## Discussion

Although our sample size was relatively small (37 useable recaptures), it was within the recapture sample size range used to generate tiger shark growth curves in other previously-published studies (e.g. N = 19, [12], N =  42, [10]). Our sample also consisted of validated measurements of growth over a wide range of tiger shark sizes, and yielded robust estimates of growth using the Francis [15] method. This method has been used previously to estimate growth of several shark species including dusky shark (*Carcharhinus obscurus*) [19], [26], shortfin mako shark (*Isurus oxyrhincus*) [27], tiger shark [13], porbeagle shark (*Lamna nasus*) [28], blue shark (*Prionace glauca*) [29], school shark (*Galeorhinus galeus*) [30], and whiskery shark (*Furgaleus macki*) [31]. In studies where both the Francis [15] and Gulland and Holt [23] methods were employed they either produced similar growth estimates [29], [30] or the Francis [15] method produced more biologically realistic growth estimates [13], [29], [32]. An important caveat of all growth models is that reliability can be compromised when recapture size range is not representative of population size ranges. The Gulland and Holt [23] method is particularly vulnerable to such shortcomings. This is apparent in the present study with the lack of recaptured very large (i.e. slowest growing) individuals resulting in the Gulland and Holt [23] model producing a smaller *L_∞_*. Although it is not possible to statistically compare the two methods, the Francis [15] method is preferred over the Gulland and Holt [23] method for Hawaii tiger shark tag/recapture data for two reasons: 1) residual plots indicate a satisfactory fit to the data, and 2) the best fit model included parameters for individual variability in growth (*v*) and measurement error (*s*), thus warranting the use of a model that can capture these potential sources of bias.

Failing to explicitly account for individual variability in growth (i.e., assuming all individuals in a population grow according to fixed parameters) and measurement error can result in biased mean growth estimates [32]–[34]. Estimates of Hawaii tiger shark individual variability in growth are similar to those produced by the Francis [18] method for western north Atlantic tiger sharks [13]. This suggests that individual variability in growth is typical for tiger sharks across their geographic range, as is also the case in other carcharhinids (e.g. Blacknose sharks, *Carcharhinus acronotus*; [35]). Although small, the best fit model also included *s* indicating that it was substantial enough to influence growth estimates. The inclusion of *s* but not *m* is common in many growth studies that use the Francis method [19].

Our results suggest tiger sharks in Hawaii grow twice as fast as previously believed (*K*  =  0.31, this study, versus *K* = 0.16 [16]), and thus exhibit growth rates consistent with those seen in several other regions (e.g. *K* = 0.27–0.46 in NW Atlantic [13]). Overall, tiger sharks are fast growing compared to other shark species in Hawaii (e.g. *K* = 0.10–0.12 for Hawaii sandbar sharks, *Carcharhinus plumbeus* [14]). This is especially true of juvenile tiger sharks which were found to grow over 100 cm year^−1^ in Hawaii, a rate comparable to that recently reported for juvenile tiger sharks in the South Atlantic (118 cm year^−1^ [36]). This rapid growth may be a strategy for reducing juvenile predation risk as has also been suggested for the rapidly-growing smalltooth sawfish, *Pristis pectinata*, which is another elasmobranch that reaches a large adult size [37]. In order to fuel their rapid growth, tiger sharks presumably eat more prey than slower-growing sharks, and may achieve this by targeting slow-moving, armored and toxic species that are infrequently exploited by other predators (e.g. [5], [6]).

Another consequence of this rapid growth is that tiger sharks in Hawaii probably reach maturity at a relatively young age. Using the minimum size of maturity estimates provided by Whitney and Crow [7] (Fig. 3), our new growth rate estimate suggests that on average female tiger sharks reach maturity by age 5 and males by age 4, with the fastest growing individuals of both sexes reaching maturity in as little as 3 years, comparable to recent estimates of minimum age of maturity for tiger sharks in the South Atlantic off Brazil (approximately 3.5 years, [36]).

Other demographic characteristics of the Hawaii tiger shark population were also consistent with those in several other regions. For example, the sex ratio among Hawaii tiger sharks was significantly skewed toward females, as has also been reported from Australia [12],[38]–[40], the Southeastern United States [41] and South Africa [11]. This female-biased ratio may indicate sexual segregation (e.g., females occupy coastal areas where most fishing effort occurred, whereas males occupy offshore habitats [42]) as *in utero* sex ratios in Hawaii are close to 1∶1 [7]. Sexual segregation is widespread in the animal kingdom [43], is seen in other carcharhinid species in Hawaii (e.g. sandbar sharks, [1], [44]) and may be driven by sex-specific environmental preferences [42].

Hawaii female tiger sharks were significantly larger on average than males, a phenomenon also evident in other locations (e.g., Queensland, Australia [38]) but not ubiquitously (e.g., males were found to be significantly larger than females in Shark Bay, Western Australia [12], [45]). The maximum observed size of tiger sharks in Hawaii (464 cm TL) is generally consistent with those reported from other regions where sharks have been empirically measured as part of scientific studies (Table 1). Tiger sharks of 400 cm TL or larger are relatively common in Hawaii, accounting for 10% of our sample, whereas those larger than 450 cm TL were very rare, (0.005% of sharks captured). One empirical study conducted in Australia reported a maximum size (550 cm TL) [40], considerably larger than any scientifically-measured tiger shark in any other location (Table 1). This Australian study had a very large sample size (4,757 sharks) compared to most others, hence had a higher probability of picking up the rare, very largest individuals in the population, than studies with sample sizes of an order of magnitude lower. One concern associated with estimating maximum size of large sharks is that the very largest individuals in the population may simply be escaping capture by straightening hooks or breaking net meshes. We do not believe this to be the case in the present study because our fishing gear was similar to that used during the Australian study which captured tiger sharks of up to 550 cm TL. Thus we conclude that tiger sharks larger than 450 cm TL are very uncommon, and only an extremely small number of individuals reach or exceed 550 cm TL. The veracity of the report of a 740 cm TL (3,110 kg) female caught off Indo-China in 1957 [8] is unclear, as it is based on an unverified, annotated photograph rather than an empirical measurement.
